# BCL11A mRNA Targeting by miR-210: A Possible Network Regulating γ-Globin Gene Expression

**DOI:** 10.3390/ijms18122530

**Published:** 2017-11-26

**Authors:** Jessica Gasparello, Enrica Fabbri, Nicoletta Bianchi, Giulia Breveglieri, Cristina Zuccato, Monica Borgatti, Roberto Gambari, Alessia Finotti

**Affiliations:** 1 Department of Life Sciences and Biotechnology, Ferrara University, 44121 Ferrara, Italy; jessica.gasparello@unife.it (J.G.); enrica.fabbri@unife.it (E.F.); nicoletta.bianchi@unife.it (N.B.); giulia.breveglieri@unife.it (G.B.); cristina.zuccato@unife.it (C.Z.); monica.borgatti@unife.it (M.B.); 2 Laboratory for the Development of Pharmacological and Pharmacogenomic Therapy of Thalassaemia, Biotechnology Center, Ferrara University, 44121 Ferrara, Italy

**Keywords:** microRNAs, miR-210, BCL11A, hemoglobin, erythroid differentiation, β-thalassemia, γ-globin

## Abstract

The involvement of microRNAs in the control of repressors of human *γ-globin* gene transcription has been firmly demonstrated, as described for the miR-486-3p mediated down-regulation of BCL11A. On the other hand, we have reported that miR-210 is involved in erythroid differentiation and, possibly, in *γ-globin* gene up-regulation. In the present study, we have identified the coding sequence of BCL11A as a possible target of miR-210. The following results sustain this hypothesis: (a) interactions between miR-210 and the miR-210 BCL11A site were demonstrated by SPR-based biomolecular interaction analysis (BIA); (b) the miR-210 site of BCL11A is conserved through molecular evolution; (c) forced expression of miR-210 leads to decrease of BCL11A-XL and increase of γ-globin mRNA content in erythroid cells, including erythroid precursors isolated from β-thalassemia patients. Our study suggests that the coding mRNA sequence of BCL11A can be targeted by miR-210. In addition to the theoretical point of view, these data are of interest from the applied point of view, supporting a novel strategy to inhibit BCL11A by mimicking miR-210 functions, accordingly with the concept supported by several papers and patent applications that inhibition of BCL11A is an efficient strategy for fetal hemoglobin induction in the treatment of β-thalassemia.

## 1. Introduction

MicroRNAs (miRNAs, miRs) are a category of conserved, short (19 to 25 nucleotides in length) RNAs that regulate gene expression by targeting specific mRNA sequences [1,2,3], causing a post-transcriptional negative control or mRNA degradation, depending on the level of base-pairing between miRNAs and target RNA sequences [1,4,5]. Since their discovery and first characterization, the number of validated microRNA sequences enlisted in databases has significantly grown [3]. Moreover, considering that a mRNA might contain in its 3′ untranslated region (3′UTR), coding DNA sequence (CDS), 5′ untranslated region (5′UTR) several sequences for miRNA recognition and that a single miRNA can bind and regulate several mRNAs, it is calculated that more than 60% of human mRNAs are targets of microRNAs [2,4]. Therefore, a great interest is concentrated on the study of validated miRNA targets. Considering the role of microRNAs, low expression of a given miRNA is expected to be linked with an accumulation of the target mRNAs; conversely, high expression of miRNAs is expected to be associated with low expression of the target mRNAs.

In this respect, increasing numbers of reports about the roles of microRNAs in erythropoiesis have been published [6,7,8,9,10,11,12,13,14,15,16,17,18]. For instance, Felli et al. identified miR-221 and miR-222 as being highly expressed in human cord blood-derived hematopoietic CD34^+^ progenitor cells [6]. MicroRNA expression profiling was also performed by Choong et al. on ex vivo differentiating erythroid cultures derived from human umbilical cord blood (UCB) CD34^+^ cells and K562 cells, with the aim of identifying miRNAs involved in erythropoiesis [7]. After comparison of stimulated UCB-derived CD34^+^ cells and K562 cells, several miRNAs were identified putatively critical for erythroid development and maturation. MicroRNAs miR-15b, miR-16, miR-22, and miR-185 were found to have strong positive correlation with the appearance of erythroid surface antigens (CD71, CD36, and CD235a) and hemoglobin synthesis, while miR-28 displayed an inverse relationship with the expression of these markers [7]. Other efforts aimed at defining erythroid-specific miRNAs were those published by Georgantas et al., who demonstrated miR-155 as a microRNA involved in the control of both myeloid and erythroid differentiation [8]. In conclusion, miRNAs have been indicated to play roles in normal hematopoiesis [6,7,8,9,10,11,12,13,14,15,16,17,18].

We have previously reported the following observations on miR-210: (a) miR-210 expression is high in erythroid precursors from β-thalassemia patients displaying high production of fetal hemoglobin (HbF) and effective response to HbF inducers; (b) miR-210 increases following mithramycin (MTH) treatment of K562 cells and human erythroid progenitors from normal and β-thalassemic subjects; (c) this increase is associated with erythroid induction and elevated expression of *γ-globin* genes; (d) an anti-miR against miR-210 interferes with the mithramycin-induced changes of gene expression [19,20]. The involvement of miR-210 in erythroid differentiation was also reported by Sarakul et al. [21], Bavelloni et al. [22] and Sawant et al. [23].

Recently, the network between microRNAs and transcription factors has been the object of several studies, confirming the fact that it can be deeply involved in the expression of HbF production in erythroid cells [24,25]. In this respect, there is a general agreement on two key issues: (a) the expression of the *γ-globin* genes is under a strong negative control which is regulated by transcription repressors [24,25,26,27,28,29,30,31,32] and (b) some of the transcription repressors are targets of several microRNAs [24,25,33,34,35,36]. For instance, transcription factors involved in transcriptional repression of the *γ-globin* genes are Oct-1 [26], MYB [27], KLF-1 [28], BCL11A [29,30] and LYAR [31,32]. The identification of microRNAs targeting mRNAs coding for these repressors (data are available for microRNAs miR-15a, miR-16-1, miR-486-3p, miR-23a/27a) [33,34,35], could be useful to develop novel approaches for the treatment of β-thalassemia [36].

The objective of the present paper was to identify possible binding of miR-210 to transcription repressors and verify its possible function. As first putative target, the BCL11A (B-cell lymphoma/leukemia 11A) was selected in consideration of the fact that it belongs to a complex (constituted also by Sox6, GATA-1, Fog-1, Mi-2/NuRD complex) [29,30] deeply involved in repression of the *γ-globin* gene transcription. In particular, the BCL11A-XL isoform has been firmly associated with high *β-globin* gene expression and low γ-globin production [29], fully sustaining the concept that BCL11A (particularly the XL-isoform) is a major transcriptional repressor of *γ-globin* gene expression.

## 2. Results

### 2.1. Presence of miR-210 Binding Sites within mRNA Sequences Coding Repressors of γ-Globin Gene Transcription: The Coding Sequence of BCL11A mRNA Contains a Putative miR-210 Binding Site

**Figure 1:**
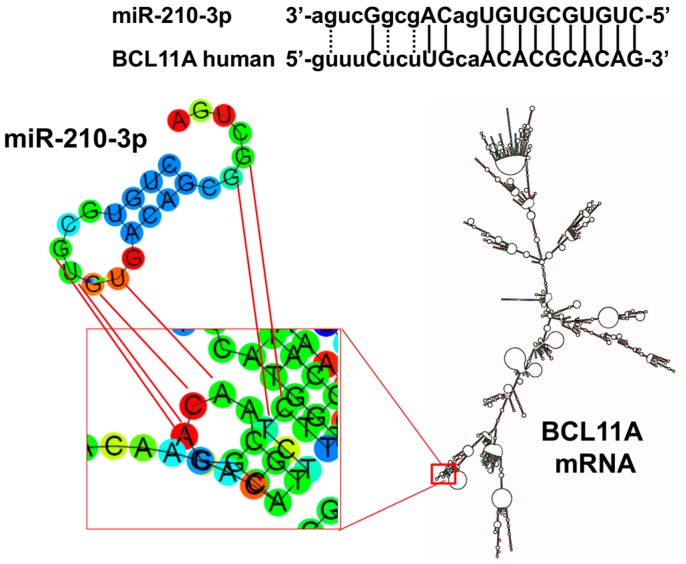
Computer-aided analysis of the possible pairing interaction between hsa-miR-210 (entry: http://www.mirbase.org/cgi-bin/mature.pl?mature_acc=MIMAT0000267) and BCL11A mRNA (http://www.ncbi.nlm.nih.gov/nuccore/NM_022893.3). The MirWalk prediction database and the RNAfold WebServer (http://rna.tbi.univie.ac.at/cgi-bin/RNAfold.cgi) were used. In the upper part of the panel, the complementarity between miR-210 and BCL11A mRNA miR-210 site is shown, in the lower part the magnification of the stem-loop secondary structure possible interactions.

Figure 1 shows the location of the putative miR-210 binding site within the BCL11A mRNA coding sequence, as found employing miRWalk 2.0, a database on predicted and validated microRNA targets (http://zmf.umm.uni-heidelberg.de/apps/zmf/mirwalk/), as well as inspection of the recent published paper by Fasanaro et al. [37], describing an integrated approach to identify miR-210 binding sites within mRNA sequences. The extent of complementarity between the human BCL11A sequences (NM_022893) with mature miR-210 is 72.7%, and is located at nucleotides 789–798, with a predicted seed length of 10, and a 0.0024 *p*-value. The location of the miR-210 binding site is within the BCL11A mRNA coding sequence and exhibits partial single stranded secondary structure interaction (RNAfold WebServer, http://rna.tbi.univie.ac.at/cgi-bin/RNAfold.cgi).

**Figure 2:**
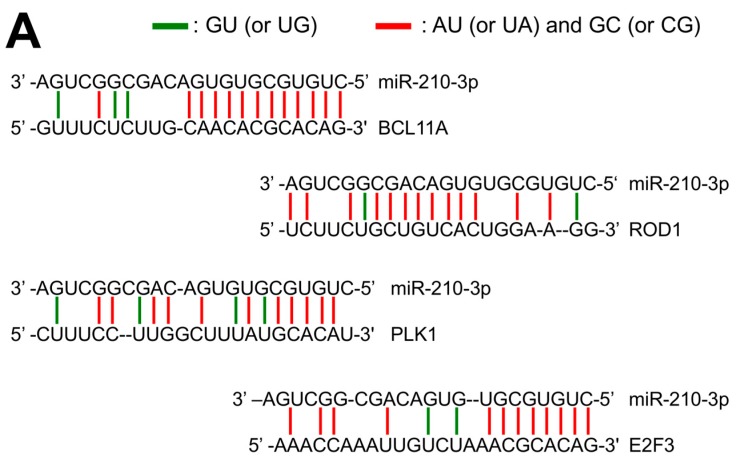

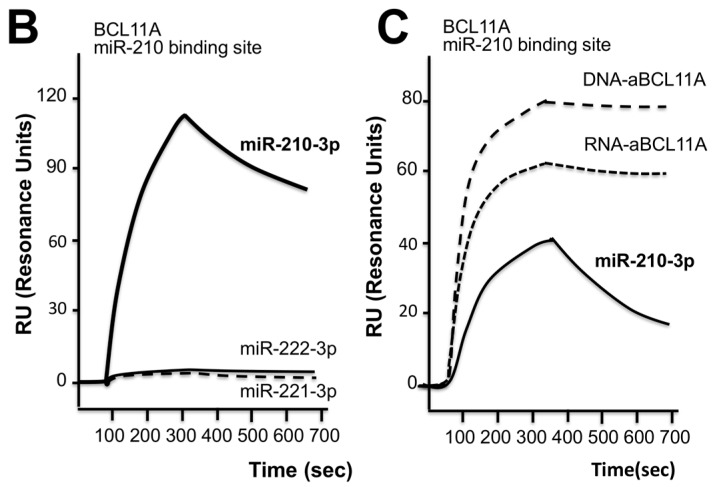
(**A**) Extent of complementarity between miR-210 and binding sites present within BCL11A mRNA (this paper) and PLK1 [38], ROD1 [39] and E2F3 [40] mRNAs; (**B**) Biacore™ X100 SPR-based analysis of the molecular interactions between miR-210, miR-221 and miR-222 and a target BCL11A sequence immobilized on a SA sensor chip; (**C**) Molecular interactions between a target BCL11A sequence immobilized on a SA sensor chip and injected miR-210 (solid line), and DNA (dotted line) and RNA (pointed line) fully complementary to the miR-210 binding site of BCL11A.

This homology (13 C-G or U-A and 3 G-U) is similar or even higher than the homology between miR-210 and other validated miR-210-target mRNAs (Figure 2A), such as PLK1 (polo-like kinase 1: 11 C-G or U-A and 4 G-U), ROD1 (Regulator of Differentiation 1: 15 C-G or U-A and 2 G-U) and E2F3 (E2F transcription factor 3: 12 C-G or U-A and 2 G-U) as elsewhere published [38,39,40].

### 2.2. Interaction of miR-210 with BCL11A Sequences Mimicking the miR-210 Binding Site of BCL11A mRNA

The ability of miR-210 to specifically interact with miR-210 binding site of BCL11A mRNA has been validated by SPR-based analysis with the Biacore™ X100 biosensor (Figure 2B). A biotinylated oligonucleotide mimicking the miR-210 binding site within BCL11A mRNA (sequence: 5′-biot-GTTTCTCTTGCAACACGCACAG-3′) was immobilized on a SA sensor chip and miR-210, miR-221 (control #1) and miR-222 (control #2) were injected obtaining the sensorgrams shown in Figure 2B. The interaction between miR-210 and the immobilized BCL11A(miR-210) oligonucleotide occurs within seconds, while miR-221 and miR-222 sequences did not bind. Interestingly, the hybrid miR-210/BCL11A(miR-210) was not fully stable, as expected from the lack of 100% complementarity between these two sequences (compare RUres with RUfin values). In fact, the extent of homology between miR-210 and the BCL11A miR-210 binding site is 59.1%. When the Biacore experiment was repeated injecting a 100% homologous RNA or DNA sequence (for nucleotide sequences see Table 1) the sensorgrams included in Figure 2C were obtained, clearly showing that in this case the generated hybrid is fully stable (RUres values almost identical to RUfin). Altogether, these data indicate that miR-210 is able to target the BCL11A miR-210 binding site, generating however, at least in these experimental conditions, unstable hybrids (unlike fully complementary DNA and RNA sequences). 

### 2.3. The miR-210 Putative Binding Sites of BCL11A Are Conserved through Molecular Evolution

**Figure 3:**
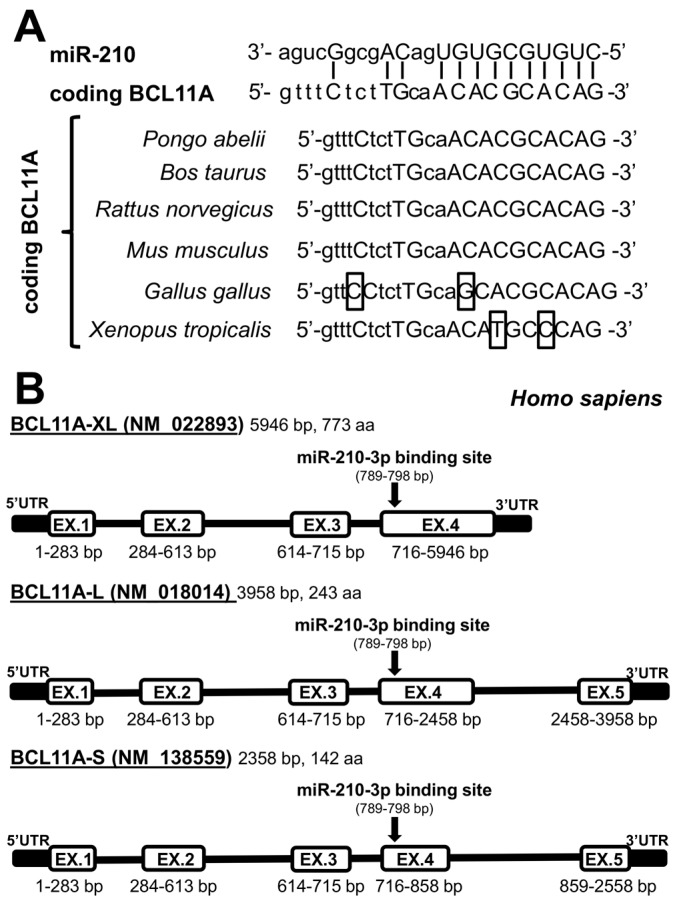
(**A**) The miR-210 binding site of BCL11A is conserved through evolution. The sequences of human (*Homo sapiens*, NM_022893.3, nucleotides 789–798), Sumatran orangutan (*Pongo abelii*, XM_002812009.2, nucleotides 709–718), bovine (*Bos taurus*, NM_001076121.1, nucleotides 787–796), mouse (*Mus musculus*, NM_016707.3, nucleotides 896–905), rat (*Rattus norvegicus*, NM_001191683.1, nucleotides 803–812), red junglefowl (*Gallus gallus*, NM_001031031.1, nucleotides 812–821), frog (*Xenopus tropicalis*, NM_001079189.1, nucleotides 592–601) are indicated. Nucleotide variations with respect to *H. sapiens* BCL11A sequences are boxed; (**B**) Location of the miR-210 binding site within the BCL11A isoforms.

The entire sequence of the *BCL11A* gene, despite being to some extent conserved, displays some variations in different species, such as human (*Homo sapiens*), Sumatran orangutan (*Pongo abelii*), bovine (*Bos taurus*), mouse (*Mus musculus*), rat (*Rattus norvegicus*), red junglefowl (*Gallus gallus*), western clawed frog (*Xenopus tropicalis*). However, as reported in Figure 3A, the BCL11A miR-210 site is highly conserved. This miR-210 binding site covers the nucleotide region 789–798 of the BCL11A mRNA, which is common to all the BCL11A isoforms (see Figure 3B). Interestingly, the nucleotide sequences surrounding the BCL11A miR-210 site exhibit some nucleotide variation. These observations clearly support the hypothesis that miR-210 plays a role in regulating the expression of the *BCL11A* gene through a direct binding to the coding sequence of BCL11A mRNA. Since the location of the miR-210 binding site occurs in a region common to all the BCL11A isoforms, we hypothesize that it could be important for post-transcriptional regulation of the general expression of *BCL11A* gene, including the overall amount of produced BCL11A-XL isoform.

### 2.4. Decrease of BCL11A-XL and Increase of γ-Globin after Transfection of a K562(BCL11A-XL) Clone with Pre-miR-210 

**Figure 4:**
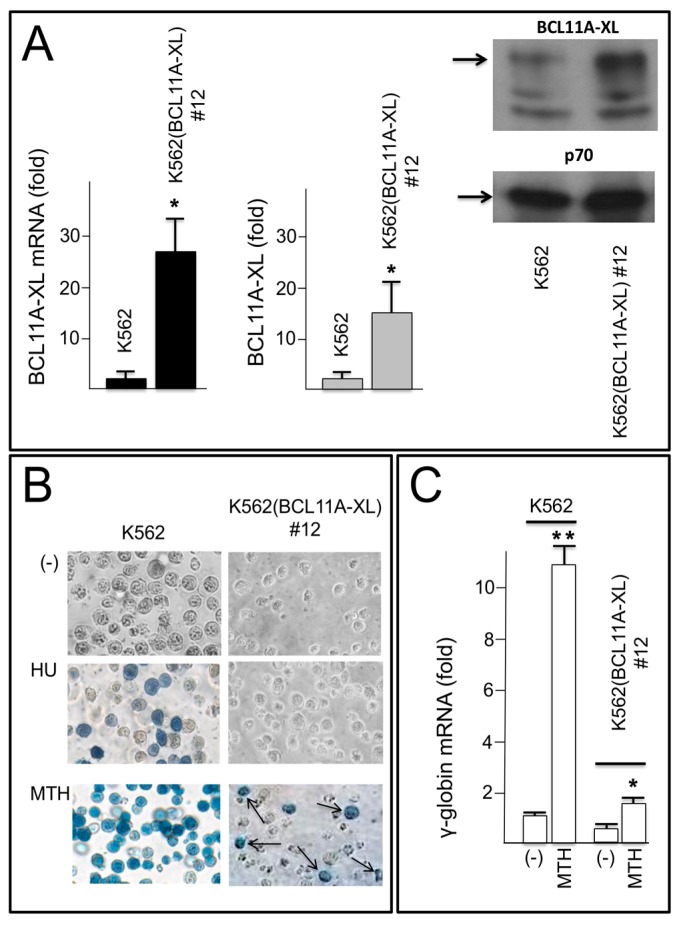
(**A**) Molecular and phenotypic characterization of the K562(BCL11A-XL) clone #12. Left: level of expression of BCL11A-XL mRNA, as determined by RT-PCR analyses of RNA isolated from the original K562 cells and the K562(BCL11A-XL) clone #12. Middle and right panels: Western blotting analysis showing differential expression of BCL11A-XL in K562 cells and K562(BCL11A-XL) clone #12, as indicated; (**B**) benzidine staining of K562 cells and K562(BCL11A-XL) clone #12 cells treated for 6 days with 175 µmol/L hydroxyurea (HU) or 30 nmol/L mithramycin (MTH). Benzidine-positive cells of K562(BCL11A-XL) clone #12 are arrowed. Pictures were obtained using 20X magnification; (**C**) level of expression of γ-globin mRNA, as determined by RT-PCR analyses of RNA isolated from the original K562 cells and the K562(BCL11A-XL) clone #12 cultured for 5 days in the absence (−) or in the presence (MTH) of 30 nM mithramycin. ** *p* < 0.01; * *p* < 0.05 (*n* = 5).

In order to obtain information on the biological effects of miR-210 on BCL11A-XL regulated genes, a K562(BCL11A-XL) clone (clone #12) was employed. The K562(BCL11A-XL) clone #12 was chosen because expresses at very high level BCL11A-XL (Figure 4A, in which RT-qPCR, left side of the panel, and Western blotting analysis, middle and right side of the panel, are shown) and it is resistant to the induction of erythroid differentiation following treatments with rapamycin (RAPA), resveratrol (RSV), butyric acid (BA) and hydroxyurea (HU) [41]. For the production of K562 cell clones with integrated copies of a BCL11A-XL expressing vector we have transfected K562 cells with the pCDNA3.1-BCL11A-XL vector, and then cloned the transfected cells by limiting dilutions. As a representative example, results of treatments of original K562 cell line and K562(BCL11A-XL) clone #12 with HU and MTH are shown in Figure 4B, demonstrating that both treatments lead to a clear increase of the proportion of benzidine-positive cells only when the K562 cell line is employed; in fact, when the K562(BCL11A-XL) clone #12 was used, a low but significant increase of the proportion of benzidine positive cells was obtained with only MTH. The production and characterization of K562(BCL11A-XL) clones have been reported elsewhere by Finotti et al. showing that untreated K562(BCL11A-XL) clone #12 expresses lower amounts of γ-globin mRNA with respect to K562 cells [41], possibly caused by the high levels of BCL11A-XL accumulation. As expected and reported in Figure 4C, this clone expresses, with respect to K562 cells, low level of *γ-globin* genes, either in the absence or in the presence of MTH, which, in any case, is able to partially rescue the *γ-globin* gene expression, suggesting that K562(BCL11A-XL) clones are able to differentiate when treated with HbF inducers inhibiting *BCL11A* expression (such as mithramycin) [41].

**Figure 5:**
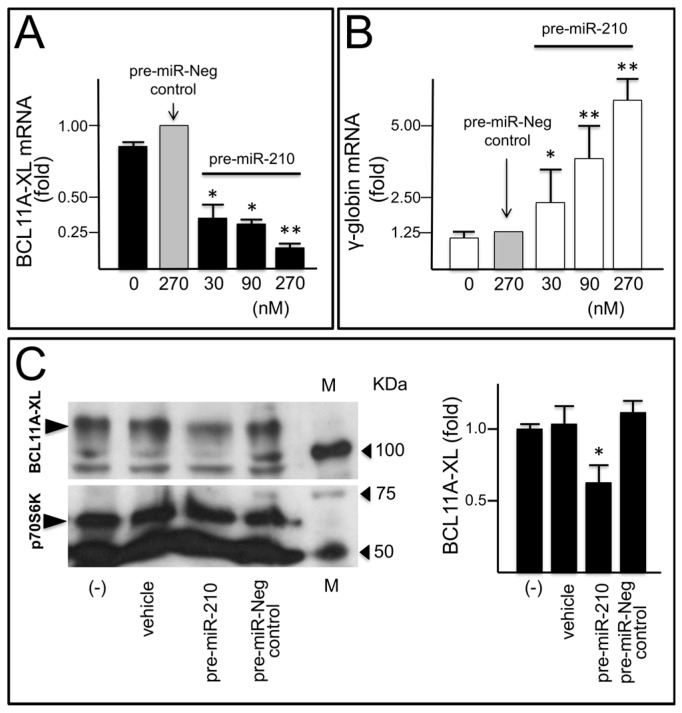
Effects of pre-miR-210 on BCL11A-XL mRNA content and *γ-globin* gene expression. (**A**,**B**) Effects of transfection with increasing amounts of pre-miR-210 and pre-miR-negative control (arrowed) on BCL11A-XL mRNA (**A**) and γ-globin mRNA (**B**); The RNA was isolated after 48 h from the transfection and the expression analyzed by RT-qPCR was expressed relative to treatment with the pre-miR-negative control. ** *p* < 0.01; * *p* < 0.05 (*n* = 3); (**C**) Western blotting (a representative experiment on the left, the analysis of three experiments on the right) performed on proteins isolated from cells treated as indicated. Ribosomal S6 protein kinase (p70S6K) was used as internal control (* *p* < 0.05; *n* = 3).

Figure 5A,B shows that transfection of K562(BCL11A-XL) clone #12 cells with pre-miR-210 leads to a sharp concentration dependent decrease of BCL11A transcripts (Figure 5A) and, at the same time, an increase of γ-globin mRNA content (Figure 5B). In order to validate these observation at the protein level, BCL11A protein production by K562(BCL11A-XL) clone #12 cells transfected with pre-miR-210, was studied and the results, presented in Figure 5C, demonstrate a significant decrease of BCL11A in transfected cells. The comparison of the effects of pre-miR-210 with those of a negative control supports the sequence-specificity of the effects. It is important to underline that the K562(BCL11A-XL) clone #12 was required for these experiments, since K562 cells do not express (or express at very low level) BCL11A (see Figure 4A).

### 2.5. Transfection of Erythroid Precursor Cells with Pre-miR-210 Leads to a Decrease of BCL11A-XL and an Increase of γ-Globin mRNA 

**Figure 6:**
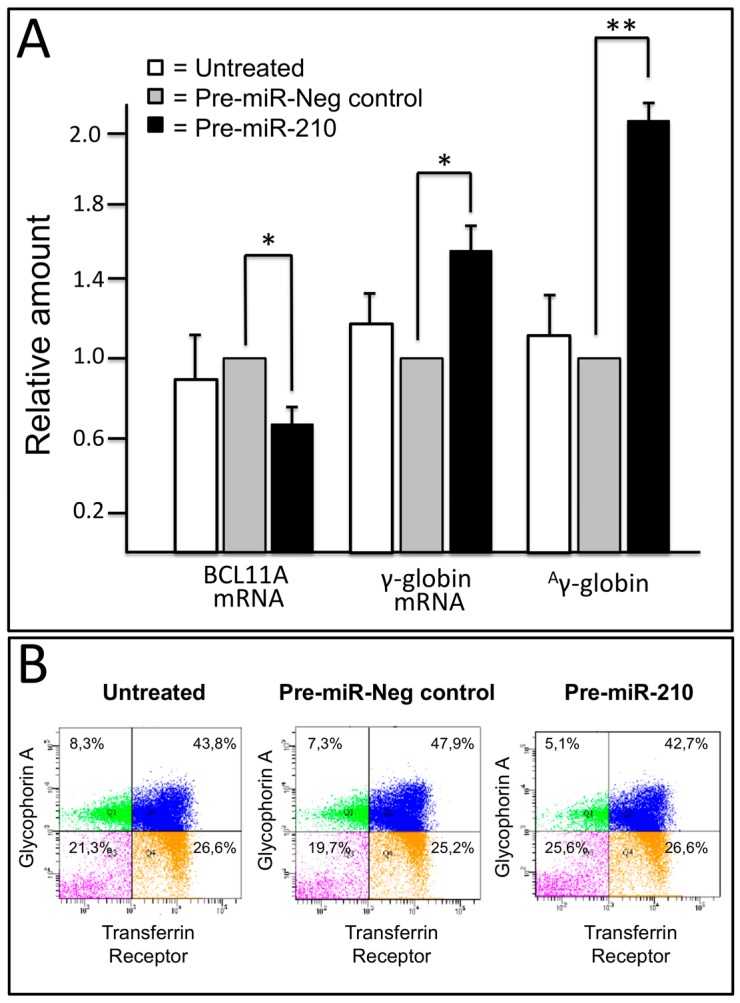
(**A**) Effects of pre-miR-210 transfection of erythroid precursor cells on BCL11A-XL mRNA content and *γ-globin* gene expression. The level of expression of BCL11A-XL mRNA and γ-globin mRNA was determined by RT-PCR analysis performed on erythroid precursor cells isolated from four β-thalassemia patients. The level of ^A^γ-globin content was determined by ELISA performed on erythroid precursor cells isolated from one β-thalassemia patient (experiment conducted in triplicate). ** *p* < 0.01; * *p* < 0.05 (*n* = 3); (**B**) FACS analysis performed on ErPCs cultured for 120 h in the absence or in the presence of the indicated treatments. In all the experiments reported in this Figure, 200 nM of pre-miR-210 and pre-miR negative control were used.

Erythroid precursor cells (ErPCs) from four β-thalassemia patients were isolated and cultured with the two-phase procedure developed by Fibach et al. [42]. Two β°-39/β°-39, one β^+^-IVSI-6/β^+^-IVSI-6 and one β°-39/β^+^-IVSI-110 were recruited, the endogenous levels of HbF were in their ErPCs within a 25–45% range. After 14 days, cells were (a) untreated, (b) transfected with 200 nM pre-miR-210 or (c) transfected with 200 nM of pre-miR negative control. After a further 120 h, BCL11A mRNA and γ-globin mRNA content were determined by RT-qPCR. The results obtained are shown in Figure 6A and clearly indicate that transfection with pre-miR-210 leads to decreased expression of BCL11A mRNA (left side of the panel) and increased γ-globin mRNA content (middle side of the panel). Moreover, ELISA testing demonstrates that fully in agreement with the repressor role of BCL11A in *γ-globin* gene expression, the decrease of *BCL11A* gene expression is accompanied by an increase of γ-globin (Figure 6A, right side of the panel).

In order to verify whether the effects of the transfection with pre-miR-210 are restricted to *BCL11A* and *γ-globin* gene expression, additional erythroid associated markers (CD71 and Glycophorin A), were studied in transfected ErPCs. The data are shown in Figure 6B and demonstrate that no changes in CD71 (Transferrin Receptor) and GPA were detectable when untreated ErPCs are compared with ErPCs transfected with pre-miR-210 and a pre-miR-negative control. In particular, it should be underlined that the treatment is not sufficient to induce the activation of the full program of erythroid differentiation.

## 3. Discussion

The management of β-thalassemia patients is mostly based on blood transfusion, chelation therapy and, alternatively, on bone marrow transplantation [24]. Recently, novel therapeutic options are explored, such as gene therapy and fetal hemoglobin (HbF) induction [24,25,26,30,36]. Despite the fact that these approaches are promising, these therapeutic strategies are at present still under deep experimental development and are the basis of only a limited number of clinical trials. For instance, hydroxyurea, the most used HbF inducer in β-thalassemia, exhibits the following limitations: (a) toxicity; (b) lack of response in about 50% of β-thalassemia patients; (c) development of drug-resistance under long-term treatments. Therefore, the validation of new HbF inducers and/or new approaches for reactivation of *γ-globin* genes are required to develop novel options in the therapy of β-thalassemia.

In this respect, the recent finding that the transcriptional regulation of *γ-globin* gene expression is under the negative control of several transcriptional repressors (including MYB, BCL11A, KLF-1, KLF-2, Sp1, LYAR) is of great interest. These evidences allow the identification of specific targets for the development of possible strategies to reactivate *γ-globin* gene expression (and consequently HbF production) by targeting repressors of *γ-globin* gene transcription. Among possible alternatives, the use of microRNA-based approaches can be proposed. In fact, the involvement of miRNAs in the control of transcriptional repressors of the human *γ-globin* genes has been firmly demonstrated. Examples are miR-15a and miR-16-1 (targeting MYB mRNA), miR-486-3p (targeting BCL11A mRNA), miR-23a (targeting KLF-2) and miR-27a (targeting Sp1).

With respect to other erythroid associated miRNAs, information is still lacking. One of the possible microRNA involved in HbF production is miR-210. We have reported that miR-210 is involved in erythroid differentiation and, possibly, in *γ-globin* gene up-regulation [19,20]. This finding has been confirmed in other laboratories by Sarakul et al. [21], Bavelloni et al. [22] and Sawant et al. [23]. For instance, a recent paper by Bavelloni et al. confirmed a six-fold increase of miR-210 following treatment of K562 cells with mithramycin. The result published by Sarakul demonstrated that miR-210 was up-regulated in K562 and β-thalassemia/HbE progenitor cells cultured under hypoxic condition. Inhibition of miR-210 expression leads to a reduction of the globin gene expression and delayed maturation in K562 and erythroid progenitor cells, indicating that miR-210 contributes to hypoxia-induced erythroid differentiation in both K562 cells and β-thalassemia/HbE erythroid progenitor cells. The possible involvement of miR-210 in HbF production is also supported by the recent communication by Sawant et al., suggesting that HbF induction by hydroxycarbamide works through miR-210 in sickle cell anemia patients [23].

In the present study, we have identified a coding sequence of BCL11A mRNA as possible target of miR-210. The following results sustain this hypothesis: (a) interactions between miR-210 and the miR-210 BCL11A mRNA site were demonstrated by SPR-based biomolecular interaction analysis (BIA) (see Figure 2B); (b) the miR-210 site of BCL11A-XL is conserved through molecular evolution, possibly indicating that these sequences play key biological functions (see Figure 2A); (c) forced expression of miR-210 leads to decrease of BCL11A mRNA and increase of γ-globin mRNA content in erythroid cells, including erythroid precursors isolated from β-thalassemia patients (see Figure 4 and Figure 5). However, the effects of the transfection with pre-miR-210 are restricted to *BCL11A* and *γ-globin* gene expression, and no changes of the other erythroid markers CD71 and GPA were detectable suggesting that the treatment with pre-miR-210 is not sufficient to induce the activation of the full program of erythroid differentiation and, therefore, should be combined with other inducers of erythroid differentiation in order to fully induce HbF accumulation. Interestingly, decreases of *BCL11A* expression was found in ErPCs from β-thalassemia patients treated with HU and MTH [41 and unpublished results] together with the expected increase of γ-globin mRNA content, further supporting the role of this transcriptional regulator and its modifiers. 

**Figure 7:**
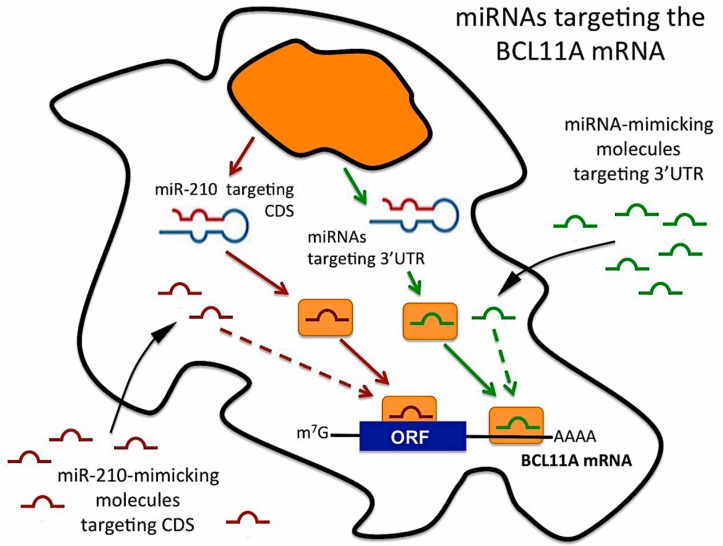
Scheme outlining the possible miRNA mimicking approach for targeting the 3′UTR and the coding sequence (CDS) of the BCL11A mRNA using miRNA mimicking molecules. Down-regulation of BCL11A gene expression will lead to up-regulation of γ-globin mRNA, as reported in Figure 4.

While most of validated miRNA/mRNA interactions involve the 3′UTR of the target mRNAs [33,34,43], functional interactions between microRNAs and coding sequences of target genes have also been reported in several studies [44,45,46]. Our study suggests that, in addition to the already reported binding of miR-486-3p to the 3′UTR sequence of the BCL11A mRNA, the coding mRNA sequence of BCL11A can be targeted by miR-210 (see the scheme reported in Figure 7). In the experimental approaches described, upregulation of *γ-globin* gene expression can be achieved by different miRNA-based approaches, including the use of pre-miRNA targeting the 3′UTR region of the BCL11A mRNA, or the use of pre-miR-210, possibly targeting the coding region. Both strategies, that might involve different miRNAs and, at least in theory, can be combined in a “multi-miRNAs therapeutic approach” might lead to extensive BCL11A down-regulation and induction of *γ-globin* gene expression, in consideration of the *γ-globin* genes repressor function of BCL11A.

In addition to the theoretical point of view, these data are of interest, in our mind, from the applied point of view, since might indicate a novel strategy to inhibit BCL11A by mimicking miR-210 functions. This is of interest, since inhibition of BCL11A is a recognized strategy for HbF induction for treatment of β-thalassemia, as suggested by several papers and patent applications. Further controls, including studies on the effects of miRNAs unable to target the BCL11A mRNA and analysis of the effects of miR-210 on BCL11A mRNA carrying a mutated and not functional miR-210 binding site should be considered for deeper validation. Moreover, a most extensive analysis on overall gene expression patterns should be considered, especially in consideration of the fact that this BCL11A site covers the nucleotide region 789–798, which is common to all the BCL11A isoforms (see Figure 3B). Finally, it should be considered that this strategy might also be applied in combination with other approaches using miRNA mimics targeting the 3′UTR of BCL11A mRNA [34], as well as other transcriptional repressors of the *γ-globin* genes, or chemical HbF inducers.

## 4. Materials and Methods 

### 4.1. Bioinformatic Analysis

Tools available online were used to identify the base pairing between the coding sequence of the target genes *MYB* [27], *KLF-1* [28], *BCL11A* [29,30], and the microRNA has-miR-210-3p. The sites are predicted by the Web Servers miRWalk, http://www.umm.uni-heidelberg.de/apps/zmf/mirwalk/index.html. To identify the RNA secondary structures at the lowest energy we used the online program RNAfold WebServer (http://rna.tbi.univie.ac.at/cgi-bin/RNAfold.cgi). A miR-210 binding site was found only in *BCL11A* sequence.

### 4.2. Biospecific Interaction Analysis (BIA) with Biacore™ X100

All procedures were performed at 25 °C and at a 5 μL/min flow rate, by using the Biacore™ X100 analytical system (GE Healthcare, Chicago, IL, USA) and HBS-EP^+^ (0.01 M HEPES, 0.15 M NaCl, 3 mM EDTA and 0.05% *v*/*v* Surfactant P20, pH 7.4) as running buffer as previously described [47,48]. In order to obtain an efficient capture of 5′-biotinylated oligonucleotide mimicking the miR-210 binding site of BCL11A mRNA onto the sensor chip, the well documented streptavidin-biotin interaction was employed. To this aim, the sensor chip SA, precoated with streptavidin, was used. After pretreatment with three 10 μL pulses with 50 mM NaOH–1 M NaCl, an injection of 10 ng/μL biotinylated oligonucleotide carrying sequences mimicking the miR-210 binding site of the BCL11A mRNA (IDT Integrated DNA Technologies, Coralville, IA, USA) was performed, followed by a wash with 50 mM NaOH. Hybridization to the immobilized oligonucleotide was performed by a 20 μL injection of 2.3 μM microRNA (miR-210, miR-221, miR-222, IDT), followed by a wash with 15 μL HBS-EP^+^ buffer and a regeneration of the sensor chip with a 5 μL pulse of 50 mM NaOH. The Biacore™ X100 Control Software and Biacore™ X100 Evaluation Software, version 2.0.1 (GE Healthcare) were used for operation and data analysis, respectively. Suitable blank control injections with running buffer were performed, and the resulting sensorgrams were subtracted from the experimental sensorgrams.

### 4.3. Ethics Statement

The use of human material was approved by the Ethics Committee of Ferrara’s District, document number 06/2013, approved on 20 June 2013. All samples of peripheral blood have been obtained after receiving written informed consent from patients and healthy donors or their legal representatives.

### 4.4. Human Cell Lines and Culture Conditions

Human leukemia K562 [38,39] and K562(BCL11A-XL) cells [41,49,50] were cultured in humified atmosphere of 5% CO_2_/air in RPMI 1640 medium (Sigma Aldrich, St. Louis, MO, USA) supplemented with 10% FBS (Biowest, Nuaillé, France), 50 U/mL penicillin and 50 μg/mL streptomycin. Mithramycin (MTH) [51,52], rapamycin (RAPA) [53], resveratrol (RVS) [54] and hydroxyurea (HU) [55] were purchased from Sigma. Treatment of cells with erythroid differentiation inducers was carried out by adding the appropriate drug concentrations at the beginning of the cultures (cells were seeded at 30,000/mL). The medium was not changed during the induction period. To determine possible antiproliferative effects cell growth was studied by determining the cell number/mL using Z1 Coulter Counter (Coulter Electronics, Hialeah, FL, USA). Erythroid differentiated cells containing hemoglobin were detected by specific reaction with a benzidine/hydrogen peroxide solution as reported elsewhere [41,51]. The final concentration of benzidine was 0.2% in 5 M glacial acetic acid and 10% H_2_O_2_. For the production of K562 cell clones with integrated copies of a BCL11A-XL expressing vector we have transfected K562 cells with the pCDNA3.1-BCL11A-XL vector, and then cloned the transfected cells by limiting dilutions and selected the clones in the presence of neomycin as reported elsewhere [41].

### 4.5. Patients and Erythroid Precursor Cultures

Erythroid precursor cells isolated from β-thalassemia patients were isolated as described elsewhere [42,53,54]. In this study two β°-39/β°-39, one β^+^-IVSI-6/β^+^-IVSI-6 and one β°-39/β^+^-IVSI-110 were recruited and their isolated ErPCs employed. Written informed consent was obtained from each patient and the samples of peripheral blood were collected just before the transfusion treatment. The Ficoll-Hypaque density gradient centrifugation was used to purify peripheral blood mononuclear cells. After isolation, the mononuclear cell layer was washed three times by adding 1× phosphate-buffered saline (PBS) solution. The pellet was then resuspended in α-minimal essential medium supplemented with 10% FBS (Celbio, Milano, Italy), 1 µg/mL cyclosporine A (Sigma Aldrich) and 10% conditioned medium obtained from supernatant of the 5637 bladder carcinoma cell line culture. This mononuclear cell suspension was grown in these culture conditions for seven days at 37 °C, under an atmosphere of 5% CO_2_ in air, with extra humidity (phase I culture) [42,49,50]. The nonadherent cells were harvested from the flask, washed in 1× PBS, and then grown in α-medium, 30% FBS, 1% deionized bovine serum albumin (BSA), 10^−5^ M β-mercaptoethanol, 1.5 mM l-glutamine, 10^−6^ M dexamethasone, and 1 U/mL human recombinant erythropoietin (EPO) (Tebu-bio, Magenta, Italy) and stem cell factor (SCF) (Inalco, Milano, Italy) (phase II culture) [42,53,54]. After seven days of cell culture phase II, the cells were treated for additional five days in the presence of 200 nM hsa-miR-210 pre-miR™ miRNA precursor (PM10516, Ambion-ThermoFisher Scientific, Waltham, MA, USA) or Pre-miR™ miRNA Precursor Negative Control #1 (Ambion-ThermoFisher Scientific).

### 4.6. Transfection Procedure 

The protocol used for transfection of pre-miRs in K562 cells and in ErPCs was reported by Ambion (Applied Biosystems, Foster City, CA, USA) and similar to that employed by Bianchi et al., taking into account the fact that K562 cells are an “in vitro” established cell line, while ErPCs are “ex vivo” primary erythroid precursors isolated from patients, needing of different experimental conditions for optimal transfection [20]; 24-well plates were used and 30,000 cell/mL (K562) or 1,000,000 cell/mL (ErPCs) were seeded. Cells transfections were performed using Lipofectamine RNAiMAX Transfection Reagent (Invitrogen, Life Technologies, Carlsbad, CA, USA) accordingly to manufacturer’s instruction, with the indicated concentrations (range 30–270 nM) of hsa-miR-210 pre-miR™ miRNA precursor (PM10516, Ambion-ThermoFisher Scientific). The transfection procedure with pre-miRs on the same cells was repeated after two days. Pre-miR™ miRNA Precursor Negative Control #1 (AM17110 Ambion-ThermoFisher Scientific) was used as negative control. 

### 4.7. RNA Extraction

Cells were isolated by centrifugation at 1500 rpm for 10 min at 4 °C, washed in PBS, lysed in Tri-reagent™ (Sigma Aldrich), according to the manufacturer’s instructions. The isolated RNA was washed once with cold 75% ethanol, dried and dissolved in diethylpyrocarbonate treated water before use.

### 4.8. Reverse Transcription and Quantitative Real-Time PCR (RT-qPCR)

For gene expression analysis 300 ng of total RNA were reverse transcribed to cDNA using the Taq-Man Reverse Transcription PCR Kit and random hexamers (Applied Biosystems) in a 50-µL reaction. Real-time-qPCR experiments were carried out using 5′ nuclease assay with primers and probes indicated in Table 2, purchased from Applied Biosystems (Applied Biosystems). Amplification of γ-globin transcripts was achieved with primers and probes purchased from IDT (forward primer 5′-TGG CAA GAA GGT GCT GAC TTC-3′; reverse primer 5′-TCA CTC AGC TGG GCA AAG G-3′; probe 5′-FAM-TGG GAG ATG CCA TAA AGC ACC TGG-TAMRA-3′). The cDNA (1 µL) was amplified for 40 PCR cycles using the Taq-Man Universal PCR Master Mix 2X (Applied Biosystems) in an CFX96 Touch Real-Time PCR Detection System (Biorad, Hercules, CA, USA). Relative expression was calculated using the comparative cycle threshold method and the endogenous controls human 18S rRNA and human RPL13A were used as normalizer genes. Duplicate negative controls (no template cDNA) were also run with every experimental plate to assess specificity and to rule out contamination. The real-time PCR reactions were performed in duplicates for both target and normalizer genes.

### 4.9. Western Blot 

Cellular extracts were obtained using Pierce RIPA Buffer (Thermo Fisher Scientific, Waltham, MA, USA) according to the manufacturer instructions and quantified using Pierce BCA Protein Assay kit (Thermo Fisher Scientific). Twenty micrograms of protein cytoplasmic extracts were denatured for 5 min at 98 °C in 1× sodium dodecyl sulfate (SDS) sample buffer (62.5 mM Tris–HCl pH 6.8, 2% SDS, 50 mM dithiothreithol (DTT) 0.01% bromphenol blue, 10% glycerol), and loaded on SDS–polyacrylamide gel in Tris–glycine buffer (25 mM Tris, 192 mM glycine, 0.1% SDS). Precision Plus Protein WesternC Standards (size range: 10–250 kDa) (Bio-Rad) was used as standard to determine molecular weight. Membrane was probed using the BCL11A primary rabbit monoclonal antibody (1:10,000) (Cat. Ab191402, AbCam, Cambridge, UK). The day after, membrane was washed and incubated with an appropriate HRP-conjugated secondary antibody (1:2000) and an HRP-conjugated anti-biotin antibody (1:1000), used to detect biotinylated protein marker, and exposed to ECL film (GE Healthcare, Little Chalfont, UK). After the stripping procedure membrane was re-probed with the primary antibody against p70S6K (Catalog No. 2708, Cell Signaling, Leiden, Netherlands), used as normalization control. Films for chemiluminescent blots were analyzed by Gel Doc 2000 (Bio-Rad) using Quantity One program to elaborate the intensity data of our specific target protein.

### 4.10. Elisa Assay 

Proteic extracts were obtained by the resuspension of cellular pellets in cold water, frozen by dry ice for 5 min and vortexed for 10 s. This step was repeated five times consecutively. Samples were finally centrifuged at 14,000× *g* for 15 min and the supernatant cytoplasmic fractions were collected and immediately frozen at −80 °C. ^A^γ-globin ELISA was performed using cell lysates with the Human Hemoglobin gamma 1 (HBg1) ELISA kit (MyBioSource, San Diego, CA, USA) according to the manufacturer’s protocol. 

### 4.11. FACS Analysis 

The erythroid differentiation status of ErPCs was investigated by studying transferrin Receptor (trfR) and glycophorin A (GPA) expression by fluorescence-activated cell sorting (FACS) analysis using anti-human CD71 fluorescein isothiocyanate (FITC)-conjugated antibody (Miltenyi Biotec GmbH, Bergisch Gladbach, Germany) and anti-human CD235a (glycophorin A) phycoerythrin (PE)-conjugated antibody (Miltenyi Biotec). According to the manufacturer’s protocol, 10 μL of antibodies were added to freshly isolated cells in 100 μL 1× PBS and 1% FBS, and cells were incubated on ice for 30 min. Cells were washed twice in 1× PBS and 1% FBS and analyzed using the BD FACS Canto II system (Becton-Dickinson, Franklin Lakes, NJ, USA). Data were analyzed using FACSDiva 8.0 software (Becton-Dickinson, Franklin Lakes, NJ, USA).

### 4.12. Statistical Analysis

All experimental data were normally distributed and presented as mean ± SD. Statistical differences between groups were compared using one-way ANOVA software. *p* Values were obtained using the Paired *t*-test of the GraphPad Prism Software. Statistical differences were considered significant when *p* < 0.05 (*), highly significant when *p* < 0.01 (**).

## Figures and Tables

**Table 1:** Oligonucleotides sequences used in Biospecific Interaction Analysis.

Name	Sequence 5′–3′	Accession No.	Location
BCL11A	biot-GTT TCT CTT GCA ACA CGC ACA G ^1^	NM_022893.3 ^2^	789–798
DNA aBCL11A	CTG TGC GTG TTG CAA GAG AAA C	NM_022893.3 ^2^	789–798
RNA aBCL11A	CUG UGC GUG UUG CAA GAG AAA C	NM_022893.3 ^2^	789–798
miR-210	CUG UGC GUG UGA CAG CGG CUG A	MIMAT0000267 ^3^	-
miR-221	AGC UAC AUU GUC UGC UGG GUU UC	MIMAT0000278 ^3^	-
miR-222	AGC UAC AUC UGG CUA CUG GGU	MIMAT0000279 ^3^	-

^1^ biot: biotinylated; ^2^ http://www.ncbi.nlm.nih.gov/nuccore; ^3^ http://www.mirbase.org.

**Table 2:** TaqMan^®^ Assays employed in Real-time quantitative PCR experiments (primers and probe sets).

Gene Name	Gene Accession Number	Assay Location	Assay ID
*BCL11A* ^1^	NM_022893.3	Exons 1–2	Hs00256254_m1
*BCL11A* ^2^	NM_022893.3	Exons 4–4	Hs00250581_s1
*18S Ribosomal RNA*	X03205.1	NA	4310893E
*RPL13A* ^3^	NM_012423.3	Exons 5–6	Hs03043885_g1

^1^ This assay was used for amplification of BCL11A transcripts in K562(BCL11A-XL) clones; ^2^ This assay was used for amplification of BCL11A transcripts in erythroid precursor cells from β-thalassemia patients; ^3^ ribosomal protein L13a.

## Abbreviations

ErPCs	Erythroid precursor cells
HPFH	Hereditary persistence of fetal hemoglobin
MTH	Mithramycin
RAPA	Rapamycin
RSV	Resveratrol
BA	Butyric acid
HU	Hydroxyurea
EPO	Erythropoietin
SCF	Stem cell factor
PBS	Phosphate buffered saline
HbF	Fetal hemoglobin
UTR	Untranslated region
CDS	Coding DNA sequence
UBC	Umbilical cord blood
BIA	Biomolecular interaction analysis
BCL11A	B-cell lymphoma/leukemia 11A
RPL13A	Ribosomal protein L13a
GPA	Glycophorin A
trfR	Transferrin Receptor
p70S6K	Ribosomal protein S6 kinase β-1
